# Supplementary material for: MetaRibo-Seq measures translation in microbiomes
Source: Nat Commun. 2020 Jun 29;11:3268. doi: 10.1038/s41467-020-17081-z (PMC7324362; doi:10.1038/s41467-020-17081-z)
Supplement: Supplementary file 10 — Supplementary Data 7 [file 41467_2020_17081_MOESM10_ESM.zip › File2/Confidence_High_Taxonomy/92293_out.krona.html]

Javascript must be enabled to view this page.

members
magnitude
magnitudeUnassigned
count
unassigned
taxon
rank

92293\_out

2

superkingdom
2
2

phylum
1239
1

1
class
186801

1
order
186802

186807
family
1

genus
79206
1


SRS023914\_contig\_number\_12706
1
species
2043169

phylum
976
1

1
200643
class

1
order
171549

815
family
1

1
genus
816


SRS147377\_contig\_number\_9793
820
species
1
